# The principle of integrality of care in the political-pedagogical projects of nursing programs

**DOI:** 10.1590/0104-1169.3381.2469

**Published:** 2014-07-22

**Authors:** Daiana Kloh, Kenya Schmidt Reibnitz, Astrid Eggert Boehs, Antônio de Miranda Wosny, Margarete Maria de Lima

**Affiliations:** 1 Doctoral student, Universidade Federal de Santa Catarina, Florianópolis, SC, Brazil. Scholarship holder from Coordenação de Aperfeiçoamento de Pessoal de Nível Superior (CAPES), Brazil; 2 PhD, Full Professor, Departamento de Enfermagem, Universidade Federal de Santa Catarina, Florianópolis, SC, Brazil; 3 PhD, Associate Professor, Departamento de Enfermagem, Universidade Federal de Santa Catarina, Florianópolis, SC, Brazil; 4 Doctoral student, Universidade Federal de Santa Catarina, Florianópolis, SC, Brazil. Scholarship holder from Conselho Nacional de Desenvolvimento Científico e Tecnológico (CNPq), Brazil

**Keywords:** Comprehensive Health Care, Nursing, Higher, Education

## Abstract

**Objective:**

to identify the political-pedagogical projects of the undergraduate nursing programs in Santa Catarina, Brazil according to the guidelines of the Ministries of Health and Education, considering the education of professionals under the principle of integrality.

**Method:**

documentary study with a qualitative approach. Nine projects were analyzed.

**Results:**

the colleges from the Southern region of Brazil are gradually incorporating the theoretical framework of the Brazilian health system and curricular guidelines, which includes the principle of integrality of care, into their political-pedagogical projects of undergraduate nursing programs. Some institutions strictly follow the curricular guidelines, while others make their own interpretation.

**Conclusion:**

most teaching institutions do not provide pedagogical support to students.

## Introduction

The creation of the Brazilian National Education Bases and Guidelines Law (LBD/96) and the Curricular Guidelines (DCN) caused changes within academia to reorient nursing education, in order to construct and strengthen the Brazilian Health System. Undergraduate programs have the autonomy to organize teaching according to the needs of their regions, breaking with the traditional idea of a minimum curricula and, at the same time, facing the challenge of organizing and incorporating the Brazilian Health System's theoretical-philosophical framework into their Political-Pedagogic Projects (PPP) and then the need to reflect upon their current pedagogical conceptions that exist in the teaching within the health field.

Integrality of care is highlighted among the fundamental principles of the Brazilian Health System. It guides policies and programmatic actions to meet the demands and needs of the population^(^ ^1^ ^)^, promoting defragmented care in which the human being is seen as a complex being immersed in different socio-cultural contexts. This principle guides the reorientation of curricular changes^(^ ^2^ ^)^ in nursing programs in order to comply with new teaching and care paradigms. In this situation of changes in the health and teaching contexts, this study's aim was to identify the political-pedagogical projects of the undergraduate nursing programs in Santa Catarina, Brazil according to the guidelines provided by the Ministries of Health and Education, examining the education of nurses according to the concept of integrality based on the pedagogical framework of Donald Schön.

Integrality of care, as one of the guiding principles of education, requires a pedagogical proposal that encourages students to reflect upon issues of healthcare practice, upon their assessment process as a tool that helps to acknowledge their deficits in order to repair such deficits, and open an opportunity to reflect upon their actions. Understanding of the learning process should be viewed as something that involves knowledge, skills and attitudes, as an opportunity to reflect upon the subjects' realities. At the same time, it enables professors and students to interact and reflect upon issues necessary for the abilities of professionals to deal with unexpected events, promoting transformations in ways of thinking and acting. For that, reflective practical thinking^(^ ^3^ ^)^ is a framework that helps support integral care within the program's pedagogical proposal because it proposes a rupture from closed models supported on a technical rationalism that no longer fits the needs of users of the Brazilian Health System.

## Method

This is a documentary study with a qualitative approach. Documentary research is a valuable source for understanding the object of study through the historical and socio-cultural contextualization it provides^(^ ^4^ ^)^.

The ten oldest Undergraduate Nursing Programs in Santa Catarina, Brazil were chosen based on considering the trajectory of these institutions and their responsibilities in the context of educating nursing professionals over time. A total of nine Undergraduate Nursing Programs scattered over the state of Santa Catarina participated in the study. Two institutions were founded in 1970, one in 1990 and six in 2000. The names of Brazilian stones and gems were used to ensure confidentiality of the participants' identities.

Data were collected from August 2011 to May 2012. The last PPP, implemented in 2009 (3), 2010 (5) and 2011 (1) were selected. Because PPPs are public documents, their full versions were either provided by the coordinators of the colleges included in the study or were available on the universities' websites. The programs' coordinators attested how recent the documents were. Data collection was guided by a form created based on the general topics of the curricular guidelines. Thematic Content Analysis^(^ ^5^ ^)^ was used to categorize the data. The following categories emerged from grouping the curricular guidelines according to their convergence: *Adherence of Political-Pedagogical Projects to the guidelines in regard to integrality of care; and Critical knots faced in education from the perspective of Integrality*. These two categories were discussed on the basis of the theoretical framework of Donald Alan Schön^(^ ^3^ ^)^ and the principle of integrality, seen as a "battle flag" of the health movement, playing the "image-objective" role, that is, serving as an indicator to signal the desirable characteristics of the health system and its practices^(^ ^6^ ^)^, as well as a health training process in the health field.

## Results

Seeking to identify the beliefs and values expressed by ethical-philosophical assumptions or principles guiding the educational process of the schools in the Southern region of Brazil, we highlight the following excerpts of the philosophical landmarks of the schools under study: *The education of the generalist nurse is one attentive to transformations taking place in society and knowledge production. It is dynamic and open to diversity, to the development of competencies and commitment to care, management, education, research and to continuous education over the course of life *(Ruby).* Health and Education are fundamental rights of all and a duty of the State; ensuring these rights implies the exercise of citizenship. Such exercise requires access to health information, involvement of the community, and the individuals' active participation. *(Amber)

Only one PPP did not clearly present its beliefs and values. The remaining philosophical landmarks are supported in concepts (conceptual landmark) that focus on the Human Being as unique, complex, singular, plural, creative, interactive being, a result of interrelations with society, environment and other human beings (Ruby, Emerald, Pyrite, Amber). Health concepts are also highlighted (Ruby, Emerald, Amber, Hematite), as well as education (Ruby, Jade, Amber), educational practices (Emerald, Hematite), transforming pedagogy (Jade), ethics (Jade, Pyrite, Fuchsite), nursing (Ruby, Emerald, Amber, Hematite), society (Ruby, Emerald, Amber, Hematite), and integral/holistic care (Ruby, Emerald, Amber, Hematite).

*The profile of graduates* is directly linked to the training of professionals with a generalist, critical and reflective profile. Knowledge and intervention in problems/situations concerning the health/disease continuum are also mentioned, showing the bio-psycho-social dimensions of its determinants (Emerald); technical-scientific and ethical-political competencies to perform healthcare activities in care practice, research, teaching and extension, as well as to develop the care and managerial model (Grenade); subjects that seek professional education in the nursing program, respecting the principles of competence, autonomy, democracy, citizenship, ethics, participation, inclusion, diversity, embracing (Jade); qualified to educate and research, based on ethical principles, specific and interdisciplinary knowledge (Ruby).

In regard to *Competencies and Skills*, six programs incorporated part or used the entire DCN/Nursing guidelines (Ruby, Pyrite, Amber, Grenade, Pink Agate, Jade). The remaining programs made their own interpretation concerning the meaning of competencies and skills and the following excerpt summarizes their understanding:* Nurses are expected to have the ability to self-guide themselves in order to provide nursing care to the individual, family, and community during the different stages of life, taking into account the context in which care is provided. Nurses are also expected to be able to do research in the nursing field, improving the knowledge of this discipline*(Esmeralda).

In regard to the curricular matrix, the programs present two remarkable characteristics: in five PPP, the matrices are organized by course, while four programs organize the matrices into modules, or have an integrative core, characterizing integrated content. 

Note that even though most of the curricula is organized by discipline and by levels of complexity, the Programs seek strategies to implement interdisciplinary integration, by grouping the phases in parallel with mandatory or optional courses and complementary activities (Grenade); by encouraging actions and theoretical reflection upon what is contained in the PPP, always with a view to put into operation the systematization of nursing care in both the hospital environment and in public health, in agreement with the basic principles of the Brazilian Health System (Pyrite); using integrative disciplines in order to connect different types of knowledge and theoretical-practical activities, as well as seek greater connection/cooperation among teaching, service and the community (Amber); by organizing the curriculum, taking into account the principle of integrality of health care (Fuchsite).

In regard to integrated curriculum, it is organized by a curricular axis based on health promotion in the Human Living Process. It assumes, as cross-sectional perspectives, health education, ethics and bioethics, connection/cooperation among research, teaching and extension, and the decision-making process (Ruby). The curricular matrices are organized based on integrative teaching cores distributed throughout the program in the construction of nursing knowledge and providing tools for nursing and consolidating the professional education process in nursing (Jade). Another program formed by integrative and thematic cores integrates content and theoretical and practical activities that enable interdisciplinary activities and a greater integration of teaching, service and community. Three basic components form the program's curricular axis: health promotion, holistic care, and management and administration (Hematite). 

In regard to the pedagogical proposal used by the programs, three PPP did not specify one (Ruby, Grenada, Emerald); four report the use of Problem-solving Methodology (Hematite, Amber, Pyrite, Jade); one PPP presented the use of Active Methodologies (Pink Agate), without specifying a specific one, and one program attempts to develop it by linking the country's social, historical, political and economic context to issues discussed worldwide with, consistency among teaching, learning, and education (Fuchsite).

All the programs implement *mandatory curricular traineeships* * in their last two phases in different sectors, such as public and/or private health facilities (primary healthcare units and hospitals), daycare, schools and nursing homes. *Complementary activities *are those, which, according to the programs' PPP, are activities developed over the course of the program and can be counted as credits to complement curricula through elective actions: participation in congresses, research and extension groups, elective courses and other avenues. 

In regard to the *Assessment Process*, the programs see it as: a gradual and cumulative assessment (Emerald, Grenade); a continuous process, mediator of learning, that is not restricted to the final results (Pyrite, Hematite, Jade); one that should enable technical and practical support to ensure students achieve intellectual self-assurance and the maturity to develop the functions related to their field of knowledge, a diagnostic and formative practice (Fuchsite). Three PPP did not present their conception of the assessment process in their programs (Pink Agate, Ruby, Amber).

Among the PPP, five have a *group to provide pedagogical support*; of these, only two (Amber, Jade) report the support that is provided to students, seeking actions that encourage personal, intellectual, professional, social and cultural development. The pedagogical support provided to professors is linked to recognition of the functioning of the programs, based on detailing/developing of objectives, competencies, skills, and methodologies of assessment to be developed by students. Philosophical, pedagogical, and specific discussions attempt to provide training to professors so that they can follow the changes and demands of an integrated curricular model, breaking with the dichotomy between theory and practice with the use of innovative pedagogical strategies.

## Discussion

The category "Adherence of Political-Pedagogical Projects to the National Curricular Guidelines from the perspective of Integrality of Care" shows that the plurality of guiding assumptions and concepts among the PPP relates to the flexibility and autonomy for schools that the curricular guidelines enable. The objective of the guidelines is to develop practices that have the impact necessary according to health indicators, meeting the needs of health system users.

It is under this view that schools in the Southern region of Brazil are gradually incorporating the theoretical philosophical framework of the Brazilian Health System into their PPP, and the curricular guidelines from the perspective of integrality of care. They lean toward a generalist education seeking the consolidation of the Brazilian Health System, comprising health and education as a right of citizens and a duty of the State in the face of a democratic society focused on the development of Human Beings. Their beliefs become concrete with the presentation of broad concepts of the Human Being, ethics, health and society. The intention to implement integral care in the educational program is apparent; there is such a tendency among the programs, characterized as a statement of good practices within the health system that refers to a set of values that is worth fighting for, because they are related to an ideal of greater justice and solidarity^(^ ^6^ ^)^. It is also linked to learning that enables students to focus on the reality and health needs of the population.

The "theoretical foundation" contained in the PPP of the colleges in the Southern region of Brazil shows a restructuring of epistemological practices, moving toward more flexible practices directed to the human being in his/her complexity. The programs tend to appreciate not only technique, but also throw themselves into the challenge of developing a *"professional artistic talent"* among students, i.e., an ability to express certain situations of practice that are unique, uncertain, conflictive, and that we cannot describe verbally but that sometimes can be expressed through observation and reflection upon actions, revealing tacit knowledge^(^ ^3^ ^)^, preparing students for life and to solve real life problems faced in professional practice.

In order to develop this *"professional artistic talent"*, the schools are assuming the challenge to develop pedagogical methodologies and curricular structures that address health problems in their integrality, considering the complexity of the individual and collective problems of users, their subjectivities in the political-social-historical-cultural context, constructing a critical-reflective awareness, the standard bearer of which is the consolidation of integrality in the program's pedagogical proposal. This is necessary to deepen the experiences of teaching integrality of care in the educational process^(^ ^7^ ^)^, considering that when education is thought of with the respective theoretical frameworks that ground integrality, it is transformed into an element that produces collective knowledge intended to promote the autonomy and emancipation of individuals for the care that is found in the different fields of nursing practice^(^ ^8^ ^)^. 

The diversity of contexts in which practice is implemented (primary healthcare units, daycare, hospitals, nursing homes, etc.), quantitative, qualitative assessment methodologies, and pedagogical support for both students and professors are all indications that schools are adhering to the proposed curricular guidelines from the perspective of integrality to ensure the continuity of care and production of knowledge relevant to the Brazilian Health System. At the same time, it means transitioning from an awareness of oneself and an awareness of the world to an awareness of oneself in the world^(^ ^9^ ^)^, in which when students understand the context and perceive themselves as being involved with the principle of integrality, at which point they can present hypotheses for the challenges presented before them and devise^(^ ^10^ ^)^ more humanized solutions. This would be an important contribution of a teaching system focused on the integrality of care, on a more just and humanized practice that is provided by professionals who are encouraged to exhibit this type of behavior.

These pedagogical frameworks are nurtured by dialogical activities, in which a reflective dialogue occurs, based on the observation of professional practice, during actions mutually undertaken by the professors and students, and becomes the focus of reflection upon practice. These reflective practices are collaborative in decision-making, greater understanding of reality, and with an exchange of knowledge and real experiences^(^ ^3^ ^)^. The professional profile under study meets the provisions of Art. 3 of the Curricular Guidelines^(^ ^11^ ^)^. Some institutions followed the guidelines, closely while others tie these guidelines to indicators such as democracy, interdisciplinary, and autonomy. There is a tendency among the programs to mention the text of the curricular guidelines together with their commitment to such content, often limiting the text itself when it not renewed/revised^(^ ^12^ ^)^.

We note that the development of competencies and abilities needs opportunities in the curriculum to be made concrete and individuals in nursing need the encouragement to act focused on integral care, with opportunities to reflect upon actions performed by the nursing professionals both within academia and when providing care. 

These opportunities should be taken into account in the curricular matrix to enable flexible, reflective and non-linear teaching; sufficient time needs to be supplied to students for them to come to know reality of practices and health actions, reflect upon them and reflect upon them again. It also requires that programs work closely with practice and service, facilitating autonomous and continuous intellectual and professional development^(^ ^11^ ^)^.

The development of curricular structures with an interdisciplinary nature is seen as a strategy to handle complex issues that involve the health-disease continuum and to respond in such a way as to broaden issues, particularly in the case of the principle of integrality, in addition to the other curricular guidelines^(^ ^13^ ^)^, such as acclimating students to the practice of service early on from the program's first semester, and the mandatory curricular traineeship concentrated in the last two phases of the programs, according to the workload proposed by the curricular guidelines. 

Accustoming students to the practice of service early on enables a process of reflection and learning about various experiences in which students become involved with the context of service, so they can make sense of information and interpret it in accordance with the relationship^(^ ^14^ ^)^ they establish with experiences. We note that the "difficulty in teaching integrality is not in conceptualizing it, but in putting it into practice, and in this respect, professionals and professors share the same anxieties and perplexity in regard to both students and patients"^(^ ^15^ ^)^, which is perhaps the biggest shortcoming of integrality. Therefore, the richness of practical experience is unlikely to be obtained in lectures, no matter how sophisticated the technologies and pedagogical proposals used, because when teaching is provided in these conventional spaces, the patient is often seen as an abstraction and there is no mutual acknowledgement between professional and user. 

The category named *Critical knots faced in education from the perspective of Integrality* indicates that the curricular guidelines, in addition to overcoming the traditional teaching model, present the development of common competencies oriented to the principles and guidelines established by the Brazilian Health System, which enables students to learn how to learn, with broad conceptions concerning health and society that result in extensive learning. The curricular guidelines also signalize the ability to develop care focused on prevention, promotion and rehabilitation^(^ ^16^ ^)^. 

Despite adherence of the programs to these recommendations, there is still certain reticence in revisiting some pedagogical conceptions that guide educational praxis in terms of pedagogical support provided to professors and students, which has become a critical knot. When pedagogical support is provided, it is often directed to a recognition of the program's functioning. Nonetheless, adherence to the curricular guidelines requires the continuous education of professors in regard to current teaching methodologies and support in relation to didactic, relational and/or communicative difficulties, preparing them to facilitate the teaching-learning process. Pedagogical support to students and professors enhances teaching and introduces integrality of care into the academic sphere, enabling those involved in the educational process to experience this process, in which all parties understand^(^ ^17^ ^)^ that education goes beyond technical ability.

Adherence to the curricular guidelines, which reinforce the principle of Integrality as the curricula's structuring axis, in addition to requiring meaningful and lasting changes in health education, also requires adherence to public health and education policies that favor, support and encourage the transformation processes^(^ ^18^ ^)^. Therefore, participation in Ministry-designed programs, such as the National Program for the Reorientation of Vocational Healthcare Training (*Pró-Saúde*) and the Program for Education for Health Work (*Pet-Saúde*), is leading teaching institutions to devise qualification strategies, approximating the reality of the service to the academia, discussing different methodological teaching proposals, and also aiding pedagogical support to professors, enabling them to understand this reorientation process of the Brazilian Health System.

In this context, understanding assessment tests as procedural and formative, inseparable from the teaching-learning process, and intended to lead to reflection and autonomy, is to enable and encourage the development of an *"artistic talent"* in students. The assessments are also intended to produce self-critiques, in the sense that students are able to identify their own progress and resistance so to define their subsequent deliberations.

To ensure change in the educational process, however, mobilization, participation, and interest on the part of the faculty members and students is required to ensure the development of a curricular design (and of a pedagogical practice) that permits learning oriented to the propositions of the Brazilian Health System, as indicated by the curricular guidelines^(^ ^11^ ^)^. 

Note that the programs are integrating applied/vocational sciences and basic sciences into their curricular traineeships. Is education based on the belief that answers to the users' problems can be found in science and technique, considering the truth to be objective and unique? Considering that the programs adhered to the curricular guidelines from the perspective of integrality, we believe the answer to this question is 'no', however, one must go to the field where practice is implemented, and learn how traineeships and theoretical-practical activities are being performed and how professors perceive such practices, that is, research in practice.

Shön does not deny the importance of teaching applied science, however, the author regards it as valid only if integrated into professional practice implemented in an environment of practical education that integrates action and reflection upon action. Considering that this process occurs in the action^(^ ^20^ ^)^ itself, in which students feel free to learn through action, they need an opportunity to turn to competent professionals who initiate them into the profession, which implies: "know how to ask for help, find the right word, see for yourselves and from your own perspective what a professional needs to be able to see"^(^ ^20^ ^)^.

In this context of constant change in nursing programs, it is the role of schools and of those involved in the educational programs to collectively identify what the critical knots are, so that, from this initial diagnosis, schools can develop strategies to overcome the challenge of training nurses committed to the principle of integrality. From this perspective, it is also a pertinent reflective process of action and upon action so that future nurses are able to develop an artistic talent as acquired during education. 

## Final considerations

Based on the curricular guidelines proposed, significant changes are observed in the context of curricula of the undergraduate nursing programs among the schools in the Southern region of Brazil. With autonomy to develop their PPP, the programs focus on their local and regional contexts and aim for generalist training. Their documents present innovation in regard to pedagogical methodologies and curricular structures based on the development of integrated curricula; the adoption of active learning-teaching methodologies that approximate students to service and the real problems experienced by health professionals and the community. 

Even though the schools in the Southern region of Brazil incorporate the philosophical theoretical framework of the Brazilian Health System and that of the curricular guidelines established for nursing programs, they need, from the perspective of incorporating the principle of integrality into their PPP, to rethink the way students overcome the difficulties faced throughout the program, considering students to be unique, singular, subject to failures and facing difficulties accruing from diverse contexts. Pedagogical support is one of the possibilities that permit the principle of integrality of care to be included in the pedagogical process and should also be provided to the nurses supervising the traineeship.

This study was limited to the analysis of documents, thus it is necessary to verify whether data presented here are in fact a reality, such as the practical application of curricula, the integration of teaching and service, and the perceptions of professors, students and managers concerning teaching.

## Footnotes

* Mandatory curricular traineeships are different from supervised activities developed throughout the course. According to the curricular guidelines "in addition to theoretical and practical content developed during nursing education, the programs must include, in their curricula, supervised internship in general and specialized hospitals, outpatient clinics, in the primary healthcare network and communities in the last two semester of the Undergraduate Nursing Program."

## References

[1] Sena RR, Silva KL, Gonçalves AM, Duarte ED, Coelho S (2008). O cuidado no trabalho em saúde: implicações para a
formação de enfermeiros. Interface Comunic, Saúde, Educ..

[2] Lima MM, Reibnitz KS, Kloh D, Ferraz F (2011). Integralidade na Atenção à Saúde e na Formação do
Enfermeiro: Análise da Literatura. Saúde Transf Soc..

[3] Schon D (2000). Educando o Profissional Reflexivo: um novo design para o
ensino e aprendizagem.

[4] Sá-Silva, Jackson R, Almeida CD, Guindani JF (2009). Pesquisa documental: pistas teóricas e
metodológicas. Rev Bras História e Ciências Sociais [Internet].

[5] Minayo MCS (2010). O desafio do conhecimento: pesquisa qualitativa em
saúde.

[6] Mattos RA, Pinheiro R, Mattos RA (2001). Os sentidos da integralidade: algumas reflexões
acerca de valores que merecem ser defendidos. Os sentidos da integralidade na atenção e no cuidado à
saúde.

[7] Andrade ZB, Costa HOG (2011). O currículo de enfermagem da UFBA e o
SUS. Rev Baiana Enferm..

[8] Machado MFAS, Monteiro EMLM, Queiroz DT, Viera NFC, Barroso MGT (2007). Integralidade, formação de saúde, educação em saúde
e as propostas do SUS: uma revisão conceitual. Cienc Saúde Coletiva..

[9] Freire P (2011). Pedagogia do oprimido.

[10] Waterkemper R, Prado ML, Medina JLM, Reibnitz KS (2013). Development of critical attitude in fundamentals of
professional care discipline: A case study. Nurse Educ Today..

[11] Ministério da Educação e Cultura (BR) (2001). Resolução CNE/CES nº 03 de 07 de novembro de 2001. Dispõe
sobre Diretrizes Curriculares Nacionais do Curso de Graduação em
Enfermagem.

[12] Lopes DN, Teixeira E, Vale EG, Cunha FS, Xavier IM, Fernandes JD (2008). Um olhar sobre as avaliações de Cursos de Graduação
em Enfermagem. Rev Bras Enfermagem..

[13] Almeida AH, Soares AH (2011). Health Education: Analysis of its Teaching in
Undergraduate Nursing Courses. Rev. Latino-Am. Enfermagem..

[14] Mabhala AM (2013). Health inequalities as a foundation for embodying
knowledge within public health teaching: a qualitative study. Int J Equity Health. [Internet].

[15] Ayres JRCM (2010). Integralidade do cuidado, situações de aprendizagem e o
desafio do reconhecimento mútuo In: Pinheiro R, Lopes TC,
organizadores.

[16] Haddad AE, Ristoff D, Passarella TM (2006). A aderência dos Cursos de Graduação em Enfermagem,
Medicina e Odontologia às Diretrizes Curriculares Nacionais.

[17] Scherer ZAP, Scherer EA (2012). Identificação dos pilares da educação na disciplina
integralidade no cuidado à saúde. Rev Esc Enferm USP..

[18] Feuerwerker Laura C. M (2013). Estratégias atuais para a mudança na graduação das
profissões da saúde. [Internet].

[19] Mitre SM, Siqueira-Batista R, Girardi-de-Mendonça JM, Morais-Pinto NM, Meirelles CAB, Pinto-Porto C (2008). Metodologias ativas de ensino-aprendizagem na
formação profissional em saúde: debates atuais. Ciênc Saúde Coletiva..

[20] Alarcão I (1996). Formação reflexiva de professores - Estratégias de
Supervisão.

